# Supplementary material for: Quality of Life and Working Conditions of Hand Surgeons—A National Survey
Source: Medicina (Kaunas). 2023 Aug 11;59(8):1450. doi: 10.3390/medicina59081450 (PMC10456479; doi:10.3390/medicina59081450)
Supplement: Supplementary file 1 [file medicina-2505653-supplementary.pdf › medicina-2505653-supplementary.pdf]

Quality of life as a Swiss hand surgeon

Dear colleagues

Thank you for taking part in our survey on working conditions and quality of life as a Swiss hand surgeon. The following survey will take about 4-6 minutes. The aim is to evaluate possible factors influencing the quality of life of Swiss hand surgeons. This survey is voluntary. The project is in the spirit of quality and relates to this year's SGH Congress.

We guarantee confidentiality and anonymity.

There are 38 questions in this survey.

Personal information

Gender
*
Please choose **only one** of the following:
- ○ Female
- ○ Male

Mandatory question

Age
*
Check all that apply
Please select one answer
Please choose **all** that apply:
- ☐ 18-29 years old
- ☐ 30-44 years old
- ☐ 45-59 years old
- ☐ 60+ years old

Mandatory question

Civil status
*
Check all that apply
Please select one answer
Please choose **all** that apply:
- ☐ Partnership
- ☐ Single
- ☐ Divorced
- ☐ Widowed

Mandatory question

What is your partner's profession? What is his/her percentage/rate of work?
*
Choose one of the following answers
Please choose **only one** of the following:
- ○ No partner
- ○ <50%
- ○ 50-80%
- ○ >80%

Make a comment on your choice here:

Do you have children?
*
Check all that apply
Please select one answer
Please choose **all** that apply:
- ☐ Yes, 1 child
- ☐ Yes, 2 children
- ☐ Yes, 3 children
- ☐ Yes, more than 3 children
- ☐ No

Mandatory question

Professional details

Position
*
Check all that apply
Please select one answer
Please choose **all** that apply:
- ☐ Resident
- ☐ Senior physician
- ☐ Leading position
- ☐ Chief position

Mandatory question

How many days of holiday do you have per year?
*
Please write your answer here:

Mandatory question

How often are you on call?
*
Check all that apply
Please select one answer
Please choose **all** that apply:
- ☐ 0-3 days/month
- ☐ 4-7 days/month
- ☐ >7 days/month
- ☐ Never

Mandatory question

What is the hospital's mandate?
*
Choose one of the following answers
Please choose **only one** of the following:
- ○ Category A
- ○ Category B
- ○ Category C
- ○ Practice
- ○ Other

Make a comment on your choice here:

Mandatory question

Have you ever requested unpaid leave? If yes, how many times? Was it granted?
*
Choose one of the following answers
Please choose **only one** of the following:
- ○ Yes
- ○ No

Make a comment on your choice here:

Working hours

According to your employment contract, how many hours are you expected to work per week?
*
Please write your answer here:

Mandatory question

How many hours do you actually work per week on average?
*
Please write your answer here:

Mandatory question

How many additional hours per week do you work in your free time?
*
Please write your answer here:

Mandatory question

Does this circumstance affect you?
(1 = very negative impact, 2 = somewhat negative impact, 3 = no impact, 4 = somewhat positive impact, 5 = very positive impact)
*
Please choose **only one** of the following:
- ○ 1
- ○ 2
- ○ 3
- ○ 4
- ○ 5

Mandatory question

Health status

Are you a smoker?
If yes, do you feel that it helps you to relax?
*
Choose one of the following answers
Please choose **only one** of the following:
- ○ Yes
- ○ No

Make a comment on your choice here:

Mandatory question

How many times a week do you consume alcohol?
If yes, do you feel that it helps you to relax?
*
Choose one of the following answers
Please choose **only one** of the following:
- ○ Never
- ○ 1-2
- ○ 3-5
- ○ >5

Make a comment on your choice here:

Do you use cannabis?
If yes, do you feel that it helps you to relax?
*
Choose one of the following answers
Please choose **only one** of the following:
- ○ Yes
- ○ No

Make a comment on your choice here:

Mandatory question

Do you use neurostimulants or amphetamines?
*
Please choose **only one** of the following:
- ○ Yes
- ○ No

How many hours do you sleep on average per night?
*
Please write your answer here:

Mandatory question

Do you suffer or have you ever suffered from chronic exhaustion, burnout or depression?
*
Choose one of the following answers
Please choose **only one** of the following:
- ○ Yes
- ○ No

Make a comment on your choice here:

Mandatory question

Satisfaction as a Swiss hand surgeon

Please rank the following areas of life in order of personal priority:
*
All your answers must be different and you must rank in order.
Please select at most 4 answers
Please number each box in order of preference from 1 to 4
- ☐ Family
- ☐ Social life / community
- ☐ Profession/Career
- ☐ Time for me (body, mind, spirit)

How would you rate your satisfaction in relation to your job/career?
*
Please choose the appropriate response for each item:

	1	2	3	4	5
**1=not at all satisfied; 5= completely satisfied**	○	○	○	○	○

How would you rate your satisfaction with your social life?
*
Please choose the appropriate response for each item:

	1	2	3	4	5
**1=not at all satisfied; 5= completely satisfied**	○	○	○	○	○

How would you rate your satisfaction with your family life?
*
Please choose the appropriate response for each item:

	1	2	3	4	5
**1=not at all satisfied; 5= completely satisfied**	○	○	○	○	○

How do you rate your satisfaction with your salary?
*
Please choose the appropriate response for each item:

	1	2	3	4	5
**1=not at all satisfied; 5= completely satisfied**	○	○	○	○	○

Do you feel that your work affects private relationships?
Comment only when you choose an answer.
Please select one answer
Please choose all that apply and provide a comment:
- ☐ Yes, because:
- ☐ No, because:

Do you feel stressed by your job during your holidays/ free time?
*
Choose one of the following answers
Please choose **only one** of the following:
- ○ Yes
- ○ No

Make a comment on your choice here:

Do you feel understood and supported by your environment regarding professional matters?
*
Choose one of the following answers
Please choose **only one** of the following:
- ○ Yes
- ○ No

Make a comment on your choice here:

Quality of life

How would you rate your quality of life in general? (1 = insufficient; 2 = moderate; 3 = good; 4 = very good; 5 = excellent)
*
Please choose the appropriate response for each item:

	1	2	3	4	5
	○	○	○	○	○

Are there occupational factors that positively influence your quality of life?
*
Choose one of the following answers
Please choose **only one** of the following:
- ○ Yes
- ○ No

Make a comment on your choice here:

Are there occupational factors that negatively influence your quality of life?
*
Choose one of the following answers
Please choose **only one** of the following:
- ○ Yes
- ○ No

Make a comment on your choice here:

Have you ever seriously thought about a career change?
*
Choose one of the following answers
Please choose **only one** of the following:
- ○ Yes
- ○ Non

Make a comment on your choice here:

Have you ever seriously considered a change of industry?
*
Choose one of the following answers
Please choose **only one** of the following:
- ○ Yes
- ○ No

Make a comment on your choice here:

Would you study medicine again? If not, what would you study?

*

❶ Choose one of the following answers

Please choose **only one** of the following:

- ◯ Yes
- ◯ No

Make a comment on your choice here:

Would you choose the same specialty again? If no, what would be your choice?

*

❶ Choose one of the following answers

Please choose **only one** of the following:

- ◯ Yes
- ◯ No

Make a comment on your choice here:

Would you be more satisfied if you could work more autonomously?

*

❶ Choose one of the following answers

Please choose **only one** of the following:

- ◯ Yes
- ◯ No

Make a comment on your choice here:

Do you aspire to a managerial position?

*

❶ Choose one of the following answers

Please choose **only one** of the following:

- ◯ Yes
- ◯ No

Make a comment on your choice here:

Which of the following would make a position at an acute hand surgery hospital more attractive?
(Please rank in descending priority)

*

❶ All your answers must be different and you must rank in order.

❶ Please select from 3 to 8 answers.

Please number each box in order of preference from 1 to 8

Please choose at least 3 items.

Please choose no more than 8 items.

- ☐ Fewer services
- ☐ Better compensation for working time
- ☐ Better compensation for services
- ☐ Part-time
- ☐ Regulation of time recording and compensation
- ☐ More autonomy
- ☐ Part-time research/ Part-time clinic
- ☐

Thank you very much for your participation and your answers.

You can now close the survey.

05-15-2022 – 12:16

Submit your survey.

Thank you for completing this survey.
